# Systematic Evaluation of *HLA-G* 3’Untranslated Region Variants in Locally Advanced, Non-Metastatic Breast Cancer Patients: UTR-1, 2 or UTR-4 are Predictors for Therapy and Disease Outcome

**DOI:** 10.3389/fimmu.2021.817132

**Published:** 2022-01-12

**Authors:** Vera Rebmann, Esther Schwich, Rafael Tomoya Michita, Lisa Grüntkemeier, Ann-Kathrin Bittner, Hana Rohn, Peter A. Horn, Oliver Hoffmann, Rainer Kimmig, Sabine Kasimir-Bauer

**Affiliations:** ^1^ Institute for Transfusion Medicine, University Hospital Essen, University of Duisburg-Essen, Essen, Germany; ^2^ Department of Medicine, Baylor College of Medicine (BCMC), Houston, TX, United States; ^3^ Department of Gynecology and Obstetrics, University Hospital Essen, University of Duisburg-Essen, Essen, Germany; ^4^ Department of Infectious Diseases, University Hospital Essen, University of Duisburg-Essen, Essen, Germany

**Keywords:** HLA-G, *HLA-G* UTR-4, breast cancer, *HLA-G* 3’UTR polymorphism, *HLA-G* UTR-2, *HLA-G* UTR-1

## Abstract

Despite major improvements in diagnostics and therapy in early as well as in locally advanced breast cancer (LABC), metastatic relapse occurs in about 20% of patients, often explained by early micro-metastatic spread into bone marrow by disseminated tumor cells (DTC). Although neoadjuvant chemotherapy (NACT) has been a successful tool to improve overall survival (OS), there is growing evidence that various environmental factors like the non-classical human leukocyte antigen-G (HLA-G) promotes cancer invasiveness and metastatic progression. HLA-G expression is associated with regulatory elements targeting certain single-nucleotide polymorphisms (SNP) in the *HLA-G* 3’ untranslated region (UTR), which arrange as haplotypes. Here, we systematically evaluated the impact of *HLA-G* 3’UTR polymorphisms on disease status, on the presence of DTC, on soluble HLA-G levels, and on therapy and disease outcome in non-metastatic LABC patients. Although haplotype frequencies were similar in patients (*n* = 142) and controls (*n* = 204), univariate analysis revealed that the UTR-7 haplotype was related to patients with low tumor burden, whereas UTR-4 was associated with tumor sizes >T1. Furthermore, UTR-4 was associated with the presence of DTC, but UTR-3 and UTR-7 were related to absence of DTC. Additionally, increased levels of soluble HLA-G molecules were found in patients carrying UTR-7. Regarding therapy and disease outcome, univariate and multivariate analysis highlighted UTR-1 or UTR-2 as a prognostic parameter indicative for a beneficial course of disease in terms of complete response towards NACT or progression-free survival (PFS). At variance, UTR-4 was an independent risk factor for a reduced OS besides already known parameters. Taken into account the most common *HLA-G* 3’UTR haplotypes (UTR-1–UTR-7, UTR-18), deduction of the UTR-1/2/4 haplotypes to specific SNPs revealed that the +3003C variant, unique for UTR-4, seemed to favor a detrimental disease outcome, while the +3187G and +3196G variants, unique for UTR-1 or UTR-2, were prognostic parameters for a beneficial course of disease. In conclusion, these data suggest that the *HLA-G* 3’UTR variants +3003C, +3187G, and +3196G are promising candidates for the prediction of therapy and disease outcome in LABC patients.

## Introduction

Locally advanced breast cancer (LABC) is characterized by large tumor sizes, lymph node spread, and an unfavorable tumor biology in the absence of distant metastasis (1). Breast cancer (BC) classification according to the expression status of the hormone receptors for estrogen and progesterone as well as the amplification of the HER-2neu gene are crucial in the management of BC therapy (2). In case chemotherapy is indicated in early and especially in LABC, a neoadjuvant therapeutic regimen is preferred. Neoadjuvant chemotherapy (NACT) aims at reducing the tumor size prior to surgical resection, improving rates of breast conserving surgery, eliminating micro-metastases, and assessing response to chemotherapy quite early (1, 2). On the basis of large meta-analyses, a more favorable outcome has been demonstrated for patients who achieve a pathological complete remission (pCR). Depending on histological subtypes, pCR rates vary (3, 4) and therapeutic options in the post-neoadjuvant setting can be adapted according to pathological response to NACT. The evaluation of success to NACT is based on clinical assessment, imaging, and histology after surgery (5). Despite major improvements in diagnostics and therapy, metastatic relapse occurs in about 20% of patients, which is often explained by early micro-metastatic spread into bone marrow, mirrored by disseminated tumor cells (DTC). There is growing evidence that the occurrence of cancer depends on various environmental factors as well as genetic susceptibilities. In this regard, gene polymorphisms have been identified as a parameter in malignancies (6). As tumor immune evasion is a crucial step in carcinogenesis, there is evidence that post-transcriptional regulation of immune-modulatory molecules is an important event in this process (3, 4, 7–9). The immune checkpoint molecule human leukocyte antigen-G (HLA-G) as membrane-anchored or soluble molecule represents a compelling factor in the context of both development and progression of cancer. HLA-G belongs to the non-classical class I molecules characterized by restricted tissue distribution, low rate of polymorphism, and immunosuppressive properties (10). Aberrant expression of HLA-G and its soluble forms is considered to support tumor immune escape (11) and often correlates with adverse clinical courses (12). However, a discrepancy between HLA-G mRNA and protein expression has often been detected in human tumors indicating post-transcriptional control of HLA-G in malignancies (13). Posttranscriptional regulation of the *HLA-G* gene occurs through nucleotide variability at the 3’ untranslated region (3’UTR) influencing HLA-G mRNA stability and/or microRNA targeting (14). Protein expression is constantly balanced by transcription levels and mRNA decay (14). The latter is dependent on the intrinsic stability of mRNA, which depends on the nucleotide sequence and the action of microRNAs (14). The *HLA-G* 3’UTR harbors several polymorphic sites (+3001C/T, +3003C/T, +3010C/G, +3027C/A, +3032C/G, +3035C/T, 3052C/T, +3092G/T, +3111A/G, +3121C/T, +3142C/G, +33177G/T, +3183A/G, +3187A/G, +3196C/G, and +3227A/G) and a 14-bp insertion/deletion (IN/DEL), which have been associated with differential HLA-G expression profiles or disease susceptibility (15–17). As these single-nucleotide polymorphisms (SNP) and the 14-bp IN/DEL are present in a short mRNA sequence and are just some nucleotides apart, it has to be considered that each polymorphic site in the *HLA-G* 3’UTR may not be independent of other polymorphic sites (14). A recent meta-analysis validated the *HLA-G* 3’UTR 14-bp IN/DEL polymorphisms as an eminent player in BC with the 14-bp IN allele assuming a protective role (6). Although the different mechanisms by which the various polymorphic sites influence HLA-G expression are still unknown, *in silico* studies revealed that several microRNAs target the *HLA-G* 3’UTR (18, 19). In this regard, HLA-G regulatory microRNAs have been identified that functionally target the *HLA-G* 3’UTR and bind with low or high affinity (13). As, for instance, binding of the key regulators depends on the presence of a Guanine at position +3142 (19), it is likely that polymorphisms in the *HLA-G* 3’UTR impact microRNA binding and thus HLA-G implications.

Here, we systematically evaluated the impact of HLA-G 3’UTR polymorphisms on disease status, on the presence of DTC, and known levels of soluble and vesicular HLA-G (20), and on therapy as well as disease outcome in 142 non-metastatic LABC patients.

## Materials and Methods

### Patients’ Characteristics

A total of 142 patients diagnosed between November 2007 and June 2012 at the Department of Gynecology and Obstetrics, University Hospital Essen, with histologically confirmed early and LABC were analyzed. Clinical characteristics of the patients are documented in **Table 1**. The median follow-up time for progression-free survival (PFS) was 66 months and that for OS was 70 months (range: 4 to 138 months). Thirty-three out of 133 patients experienced a disease relapse and 21/121 patients died of BC. The patients’ eligibility criteria, NACT treatment regimens, and definition of NACT response have already been described in detail elsewhere (20).

**Table 1:** Patient characteristics.

		Pre-NACT	Post-NACT
		*n* (%)	*n* (%)
**Total**	142		
**Age, years**	Median: 50 (18–83)		
**Follow-up, months**	71 (4–138)		
**Menopausal status**	Premenopausal	67 (47.2)	
	Perimenopausal	19 (13.4)	
	Postmenopausal	56 (39.4)	
**Nodal status****^a^**	Node-negative (c/pN-)	71 (50.4)	86 (61.9)
	Node-positive (c/pN+)	70 (49.6)	53 (38.1)
**Tumor size****^a^**	≤ c/pT1a-c	36 (25.7)	49 (35.5)
	c/pT2	84 (59.2)	40 (29.0)
	> c/pT2	20 (14.1)	49 (35.5)
**Tumor grading****^a^**	G1	9 (6.5)	
	G2	66 (47.5)	
	G3	64 (46.0)	
**Histological finding****^a^**	Ductal	103 (73.6)	
	Lobular	18 (12.9)	
	Other	19 (13.6)	
**Tumor subtype**	ER-, PR-, HER2-	26 (18.3)	
	ER-, PR-, HER2+	10 (7.0)	
	ER+/PR+, HER2-	77 (54.2)	
	ER+, PR+, HER2+	29 (20.4)	
**NACT regimen**	CTX	96 (68.1)	
	CTX + Trastuzumab	22 (15.6)	
	CTX + Trastuzumab + Lapatinib	8 (5.7)	
	CTX + Avastin	5 (3.5)	
	HTX	10 (7.1)	
**DTC****^a^**	Pos/total	31/117	
**Pathological response****^a^**	Complete response		29 (21.2)
	Partial response		98 (71.5)
	No response		10 (7.3)
**Survival (OS)****^a^**	Alive		122 (85.9)
	Dead		20 (14.1)
**Progression-free survival (PFS)**	Alive		109 (76.8)
	Relapsed		33 (23.2)

a Clinical parameters could not be determined for all patients; CTX, chemotherapy; DTC, disseminated tumor cells; ER, estrogen receptor; HTX, anti-hormonal therapy; NACT, neoadjuvant chemotherapy; PR, progesterone receptor.

A total of 204 healthy controls (HC), namely, 122 females and 82 males, served as a control panel for genotyping. At time of blood sampling, the median age of HC was 52 years, ranging from 21 to 73 years. Written informed consent was obtained by all participants, and the study was approved by the Local Ethics Committees (Essen 05-2870 and 17-7495) and was performed according to the Declaration of Helsinki.

### Detection Evaluation of DTC

DTC were analyzed as described before (21). In short, DTC were analyzed by immunocytochemistry using the pan-cytokeratin antibody A45-B/B3.

### Isolation of Extracellular Vesicles and Assessment of HLA-G levels

Extracellular vesicles (EV) were isolated from plasma samples by the use of ExoQuick™ (SBI Systems Bioscience Inc., Mountain View, VA, USA) as described previously (20). Plasma samples and the corresponding EV suspensions were diluted 1:2 in PBS and analyzed for the content of HLA-G molecules as described before (20, 22). For quantification of HLA-G, purified HLA-G1 (23) served as standard reagent. Levels were determined by four-parameter curve fitting. ELISA detection limit of HLA-G was 0.25 ng/ml.

### *HLA-G* 3’UTR Analysis

After genomic DNA extraction from Cytospin preparations containing single-cell suspensions derived from liquid biopsies using the QIAamp DNA Blood Mini Kit (Qiagen, Hilden, Germany) according to manufacturer’s instructions, *HLA-G* 3’UTR typing was performed by polymerase chain reaction (PCR) as previously described (24, 25). To verify differences regarding haplotype frequencies among the patient cohort (*n* = 142) and controls (*n* = 204), a residuals analysis was performed.

### Statistical Analysis

Allele and genotype frequencies of polymorphic sites were calculated by using Fisher’s exact test. Contribution of haplotypes and allelic variants to clinical parameters was evaluated by either two-sided Chi-square test or Fisher’s exact test. Stepwise multivariate Cox regression according to proportional hazards assumption or binomial logistic regression was used to identify prognostic factors for PFS/OS or therapy outcome towards NACT, respectively. *p*-values <0.05 were considered statistically significant. Statistical analyses were performed by using SPSS 25.0 software (SPSS Inc., Chicago, IL, USA) or GraphPad Prism V8.4 software (GraphPad Software, San Diego, CA, USA). OS and PFS analyses were assessed by the *survminer* package implemented in the R package version 0.4.3 using the method of Kaplan–Meier and compared using log-rank test (https://CRAN.R-project.org/package=survminer).

## Results

### *HLA-G* 3’UTR Haplotype Analysis in LABC Patients and HCs

To determine *HLA-G* 3’UTR haplotypes, 15 SNPs of the *HLA-G* 3’UTR were sequenced and analyzed in 142 well-defined, non-metastatic LABC patients and 204 healthy donors (HC). Haplotype analysis revealed 8 out of 18 haplotypes with a frequency >1%. As expected (19), UTR-1 (30% and 35%) and UTR-2 (31% and 30%) displayed the most frequent haplotypes in both LABC patients and HC, respectively, accounting for roughly 60% of the haplotypes (Additional File 1). Of note, examination of the haplotype or genotype distribution revealed that distribution of both the haplotypes and genotypes was similar among LABC patients and HC and did not reach significance (Additional File 2).

### *HLA-G* 3’UTR Haplotypes UTR-4 and UTR-7 Associate With High or Low Tumor Burden Pre-NACT in LABC Patients

**Figure 1:**
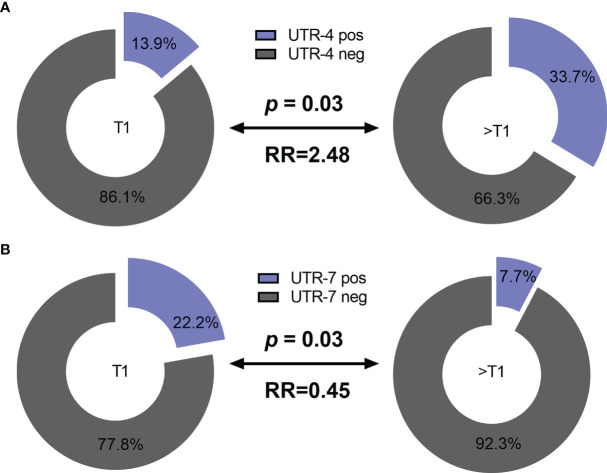
Association of the *HLA-G* 3’UTR haplotype UTR-4 and UTR-7 carrier status with tumor size pre-NACT in LABC patients. Higher frequencies of UTR-4 haplotype were observed in LABC patients with tumor size >T1 compared to patients with stage T1 pre-NACT **(A)**. Frequencies of UTR-7 haplotypes were significantly reduced in UTR-7-positive patients compared to UTR-7-negative ones **(B)**. Blue or gray color indicated frequencies of UTR-4/7-positive and UTR-4/7-negative patients, respectively. RR: relative risk.

Concerning the clinical status of LABC patients, presence of UTR-4 was positively associated with a tumor size >T1 (*p* = 0.03, relative risk [RR]: 2.48, 95% confidence interval [CI]: 1.11–5.9) as 33.7% (35/104) of patients having a tumor size >T1 carried UTR-4, whereas only 13.9% (5/36) of patients with T1 were positive for UTR-4 (**Figure 1A**). In contrast to UTR-4, presence of UTR-7 seemed to be associated with lower tumor stage (*p* = 0.03, RR: 0.45, 95% CI: 0.27–0.87): 22.2% (8/36) of the LABC patients with T1 carried UTR-7, while only 7.7% of patients with *T* > 1 were positive for this haplotype (**Figure 1B**). None of the *HLA-G* 3’UTR haplotypes were associated with high/low tumor burden or other clinical disease status pre-NACT.

### *HLA-G* 3’UTR Haplotype UTR-4 and UTR-3/7 Associate With the Presence or Absence of DTC Pre-NACT in LABC Patients

Next, we asked whether *HLA-G* 3’UTR haplotypes were associated with the presence or absence of DTC pre-NACT in LABC patients. Fifteen out of 31 LABC patients (48%) with DTC pre-NACT carried the UTR-4 haplotype, whereas only 22 out of 86 patients (22%) without detectable DTC were positive for UTR-4 (**Table 2**, *p* = 0.02, RR: 2.03, 95% CI: 1.13–3.65). At variance to UTR-4 haplotype, the UTR-3 and UTR-7 haplotype were significantly (*p* = 0.01 and *p* = 0.02, respectively) associated with the absence of DTC pre-NACT (**Table 2**, RR: 0.11, 95% CI: 0.00–1.70 and RR: 0.13, 95% CI: 0.00–1.99, respectively). None of the remaining *HLA-G* 3’UTR haplotypes were associated with the presence or absence of DTC.

**Table 2:** Association of *HLA-G* 3**’**UTR haplotypes with the presence of DTC pre-NACT in LABC patients.

		DTC pos	DTC neg	*p*	RR (95% CI)
		*n* (%)	*n* (%)		
**UTR-4 pos**		15 (48)	22 (26)	0.02	2.03 (1.13–3.65)
**UTR-4 neg**		16 (52)	64 (74)		
**UTR-3 pos**		0 (0)	15 (17)	0.01	0.11 (0.00–1.70)
**UTR-3 neg**		31 (100)	71 (83)		
**UTR-7 pos**		0 (0)	13 (15)	0.02	0.13 (0.00–1.99)
**UTR-7 neg**		31 (100)	73 (85)		

### *HLA-G* 3’UTR Haplotype UTR-7 Associates With Increased Levels of Soluble HLA-G Molecules in LABC Patients

**Figure 2:**
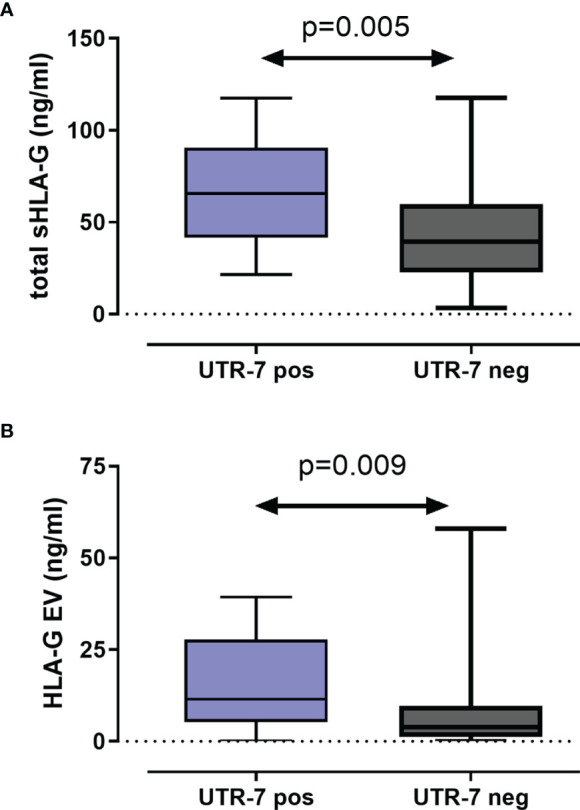
Association of the *HLA-G* 3’UTR haplotype UTR-7 with increased levels of soluble HLA-G molecules pre-NACT in LABC patients. Levels of total amount of soluble HLA-G [sHLA-G; **(A)**] and of vesicular HLA-G [HLA-EV; **(B)**] were increased in UTR-7 carrier (blue) compared with UTR-7-negative patients (gray). Data are presented as median with the minimum and maximum. Statistical significance was determined by Mann–Whitney test.

For 116 LABC patients, the levels of total amount of soluble HLA-G (sHLA-G) and of vesicular HLA-G (HLA-GEV) were available (20). To investigate the impact of *HLA-G* 3’UTR haplotypes on pre-NACT sHLA-G/HLA-GEV levels, the patients were stratified according to the presence and absence of a certain haplotype. As shown in **Figures 2A, B**, pre-NACT levels of sHLA-G as well as HLA-GEV were significantly higher in UTR-7-positive LABC patients (*p* = 0.009; *n* = 14) than in UTR-7-negative ones (*n* = 102). None of the remaining *HLA-G* 3’UTR haplotypes were associated with sHLA-G/HLA-GEV.

### *HLA-G* 3’UTR Haplotype UTR-1 Is an Independent Prognostic Co-Variate for Complete Response to NACT in LABC Patients

**Figure 3:**
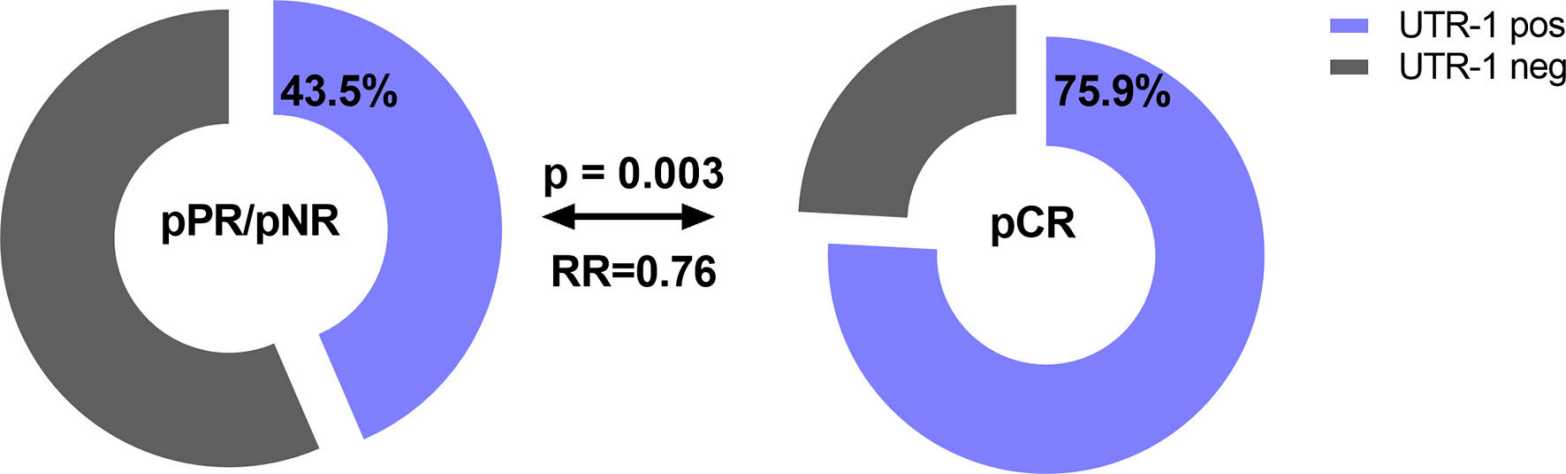
Association of the *HLA-G* 3’UTR haplotype UTR-1 carrier status with pathological complete response to NACT in LABC patients. Higher frequencies of UTR-1 haplotype were observed in LABC patients with pCR compared to patients with insufficient therapy response. Clinical data were available for all patients. Blue or gray color indicated frequencies of UTR-1-positive and UTR-1-negative patients, respectively. pCR: pathological complete remission, pPR: pathological partial response, pNR: pathological no response, OR: odds ratio.

Concerning therapy outcome, LABC patients were stratified into patients with pathological complete response (pCR, *n* = 29) and in patients with pathological partial response or with no response (pPR/pNR, *n* = 108). The presence of UTR-1 in LABC patients seemed to be a beneficial factor for the response to NACT (*p* = 0.003, RR: 0.76, 95% CI: 0.02–1.28): 75.9% (22/29) of the LABC patients with pCR to NACT carried UTR-1, while the response to NACT was insufficient in only 43.5% (47/108) of LABC patients positive for this haplotype (**Figure 3**). None of the remaining *HLA-G* 3’UTR haplotypes were associated with the response to NACT. Multivariate analysis including age (<60 vs. >60), menopausal status (premenopausal vs. peri/postmenopausal), pre-NACT nodal status (N0 vs. >N0), pre-NACT tumor size (T1 vs. >T1), NACT regimen (CTX vs. CTX + Trastuzumab vs. CTX + Trastuzumab + Lapatinib, vs. CTX + Avastin vs. HTX), UTR-1 haplotype carrier status and levels of vesicular HLA-GEV (<15.8 vs. >15.8 ng/ml (20), revealed that the UTR-1 haplotype carrier status was exclusively an independent prognostic factor correlating with pCR remission in LABC patients (*p* = 0.003; HR: 0.221; 95% CI: 0.081–0.604; *n* = 110).

### *HLA-G* 3’UTR Haplotypes UTR-2 and UTR-4 Are Independent Prognostic Co-Variates for Beneficial Progression-Free or Inferior Overall Survival of LABC Patients

**Figure 4:**
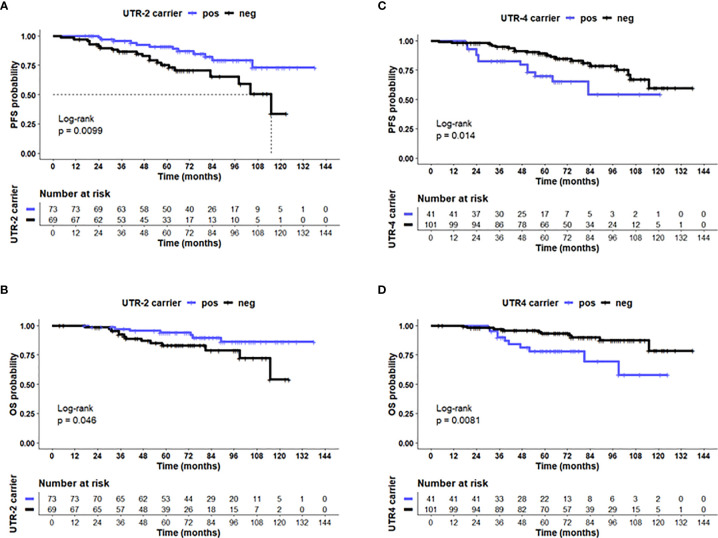
UTR-2 and UTR-4 haplotypes of the *HLA-G* gene are associated with PFS and OS in LABC patients. Kaplan–Meier plot analysis combined with log-rank test revealed that LABC patients carrying the **(A)** UTR-2 haplotype of the HLA-G 3’UTR had a significantly improved PFS **(B)** compared with UTR-2-negative patients. UTR-4-positive patients had a reduced PFS **(C)** and OS **(D)** compared to UTR-4 patients. The blue line indicates patients positive for either the **(A, B)** UTR-2 or **(C, D)** UTR-4 haplotype, whereas the black line illustrates the equivalent negative patients. Dotted line reveals median survival time where applicable. Tables under Kaplan–Meier plots show corresponding numbers at risk.

Concerning disease outcome, Kaplan–Meier probabilities of PFS (*p* = 0.01; log-rank Hazard Ratio [HR]: 0.41, 95% CI: 0.20–0.82) were significantly prolonged for patients carrying UTR-2 haplotype with undefined median PFS time compared with patients being UTR-2 negative with a median PFS of 115 months (**Figure 4A**). Similarly, the presence of UTR-2 haplotype in LABC patients showed an improved OS in comparison to UTR-2-negative ones (*p* = 0.046, log-rank HR: 0.40, 95% CI: 0.17–0.98; **Figure 4B**). In contrast to UTR-2-positive patients, combined Kaplan–Meier analysis and log-rank testing revealed that UTR-4-positive LABC patients had a significantly deteriorated PFS (*p* = 0.014, HR: 2.31, 95% CI: 1.01–5.27; **Figure 4C**) as well as OS (*p* = 0.0081, HR: 3.01, 95% CI: 1.11–8.45, **Figure 4D**) compared to UTR-4-negative LABC patients.

The UTR-1, UTR-2, and UTR-4 carrier status, the age (<60 vs. >60), menopausal status (premenopausal vs. peri/postmenopausal), post-NACT nodal status (N0 vs. >N0), post-NACT tumor size (≤T1 vs. >T1), Triple-Negative Breast Cancer (TNBC, yes vs. no), pathological response (pCR vs. pPR/NR), and levels of vesicular HLA-GEV (<15.8 vs. >15.8 ng/ml) were enrolled as co-variates in the multivariate analysis for PFS and OS. The optimal HLA-GEV cutoff value for PFS and OS was defined earlier (20). Among these co-variates, the UTR-2 haplotype appeared to be exclusively an independent factor indicative for a beneficial disease outcome in terms of PFS (*p* = 0.007, HR: 0.3 95% CI: 0.1–0.7), while a positive post-NACT nodal status (*p* = 0.009, HR: 29, 95% CI: 1.3–6.7), HLA-GEV levels >15.8 ng/ml (*p* = 0.003, HR: 4.0, 95% CI: 1.6–10.1), and a TNBC tumor subtype (*p* = 0.021, HR: 3.0, 95% CI: 1.2–7.6) were predictors for a reduced PFS (**Table 3A**). At variance to UTR-2, the UTR-4 haplotype was an independent prognostic risk factor for an inferior OS (*p* = 0.005, HR: 4.9, 95% CI: 1.6–15.1) besides a positive post-NACT nodal status (*p* = 0.015, HR: 4.3, 95% CI: 1.3–14.2, **Table 3B**).

**Table 3A:** Multivariate analysis to predict progression-free survival in LABC patients.

Co-variate		*N* = 111^a^	*p*-value	HR (95% CI)
**UTR-1**	Pos	52	0.349	0.7 (0.3–1.6)
	Neg	59		
**UTR-2**	Pos	61	**0.007**	**0.3 (0.1**–**0.7)**
	Neg	50		
**UTR-4**	Pos	37	0.267	1.7 (0.7–4.6)
	Neg	74		
**Age, years**	<60s	79	0.280	1.5 (0.2–1.7)
	>60	32		
**Menopausal status**	Premenopausal	49	0.421	1.5 (0.5–4.5)
	Peri/postmenopausal	62		
**Post-NACT tumor size**	≤T1	71	0.691	0.8 (0.3–2.0)
	>T1	40		
**Post-NACT nodal status**	N0	72	**0.009**	**2.9 (1.3**–**6.7)**
	>N0	39		
**TNBC**	Yes	22	**0.021**	**3.0 (1.2**–**7.6)**
	No	89		
**Therapy response**	pCR	102	0.222	2.7 (0.5–14.6)
	pPR/NR	9		
**HLA-GEV (ng/ml)**	<15.8	96	**0.003**	**4.0 (1.6**–**10.1)**
	>15.8	15		

**Table 3B:** Multivariate analysis to predict overall survival in LABC patients.

Co-variate		*N* = 111^a^	*p*-value	HR (95% CI)
**UTR-1**	Pos	52	0.600	1.3 (0.4–4.7)
	Neg	59		
**UTR-2**	Pos	61	0.440	0.6 (0.1–2.3)
	Neg	50		
**UTR-4**	Pos	37	**0.005**	**4.9 (1.6**–**15.1)**
	Neg	74		
**Age, years**	<60s	79	0.483	0.6 (0.1–2.6)
	>60	32		
**Menopausal status**	Premenopausal	49	0.535	1.6 (0.4–7.1)
	Peri/postmenopausal	62		
**Post-NACT tumor size**	≤T1	72	0.939	1.0 (0.3–3.6)
	>T1	39		
**Post-NACT Nodal status**	N0	71	**0.015**	**4.3 (1.3**–**14.2)**
	>N0	40		
**TNBC**	Yes	22	0.070	3.0 (0.9–10.3)
	No	89		
**Therapy response**	pCR	102	0.222	2.7 (0.5–14.6)
	pPR/NR	9		
**sHLA-GEV (ng/ml)**	<15.8	96	0.441	1.9 (0.3–10.3)
	>15.8	15		

a Clinical parameters could not be determined for all patients; NACT, neoadjuvant chemotherapy; TNBC, Triple-Negative Breast Cancer; pCR, pathological complete remission; NR, no response; pPR, pathological partial remission; HLA-GEV, vesicular-bound soluble HLA-G. Significant p-values are shown in bold.

### *HLA-G* 3’UTR SNP Variants +3187G, +3196G, and +3003C as Distinct Features of UTR-1/2, and UTR-4

**Figure 5:**
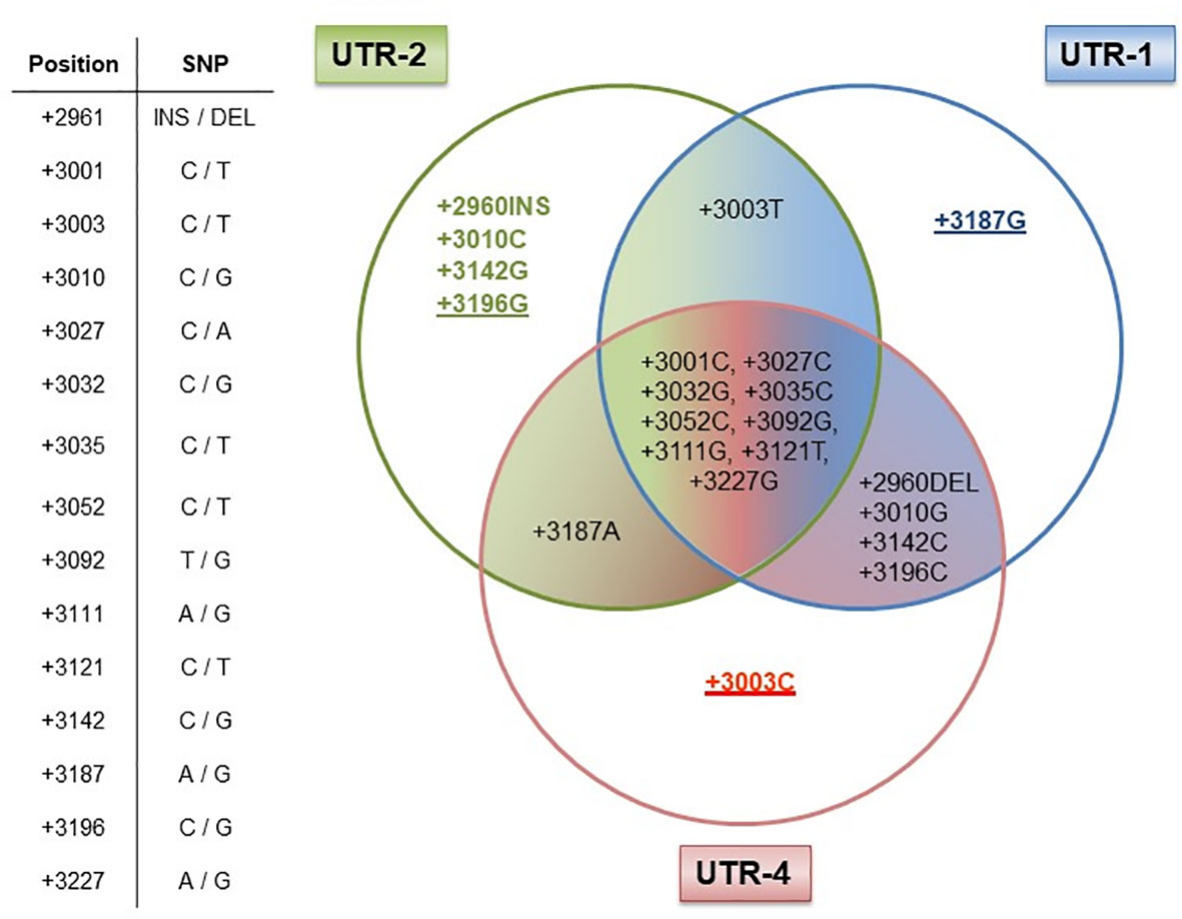
Dissection of the *HLA-G* 3’UTR haplotypes UTR-1, UTR-2, and UTR-4. Table shows the positions of the 15 single-nucleotide polymorphisms (SNP) with the HLA-G 3’UTR. UTR-1 (blue), UTR-2 (green), and UTR-4 (red) share 9 out of 15 SNP as shown in the intersection, while six SNP were different (+2960INS/DEL, +3003C/T, +3010G/C, +3142C/G, +3187A/G, and +3196C/G) among these UTR haplotypes. Underlines in allelic variants (+3187G, +3196G, and +3003C) represent the unique feature of the UTR-1, UTR-2, or UTR-4 haplotype in our patient cohort. The allelic variants +2960INS, +3003T, +3010C, and +3142G of the UTR-2 haplotype can also be found in other HLA-G 3’UTR haplotypes.

Next, we elucidated whether certain allelic variants are responsible for the antagonistic effects observed for the UTR-1/UTR-2 and UTR-4 haplotypes in LABC patients. Therefore, we analyzed the arrangement of the 15 SNP for the UTR-1, UTR-2, and UTR-4 haplotypes regarding differences and consensus in the most common 3’UTR haplotypes (UTR-1 to UTR-7 and UTR-18) in a Venn diagram (**Figure 5**). Obviously, the +3196G variant was unique for the UTR-2, whereas the +3187 variant was a distinctive feature of the UTR-1 haplotype. Thus, these variants seem to be of prognostic parameters for a beneficial course of disease. At contrast, the deduction of UTR-4 to a specific SNP revealed the +3003C variant as a unique feature, which seemed to be responsible for a detrimental disease outcome of LABC patients.

## Discussion

Various studies have suggested that the *HLA-G* 3’UTR may act as a predictor for the genetic predisposition of an individual to certain immune-mediated diseases (6, 24, 26–33). Thus, we evaluated the impact of *HLA-G* 3’UTR variants arranged as *HLA-G* 3’UTR haplotypes on the clinical status and outcome of non-metastatic LABC patients. Indeed, our study demonstrates the following: (i) Although HC and LABC patients did not differ in their UTR haplotypes’ frequencies, the UTR-4 haplotype was associated with unfavorable disease status regarding tumor size and the presence of DTC, whereas UTR-7 or UTR-3 were related to low tumor sizes, absence of DTC, and high soluble HLA-G levels, respectively. (ii) Concerning therapy and disease outcome, univariate and multivariate analysis displayed UTR-1 or UTR-2 as prognostic parameters indicative for a favorable course of disease in terms of complete response towards NACT or PFS. At variance, UTR-4 was an independent risk factor for an inferior OS besides already known clinical parameters. (iii) Taken into account the most common *HLA-G* 3’UTR haplotypes (UTR-1 to UTR-7, UTR-18) with haplotype frequencies > 1% (Additional file 1), the attribution of the UTR-1/2/4 haplotypes to specific SNPs revealed that the +3003C variant, unique for UTR-4, seems to support an adverse disease outcome, whereas the +3187G and +3196G variants, unique for UTR-1 or UTR-2, are prognostic parameters for a beneficial course of disease.

At variance to our study, in which all *HLA-G* 3’UTR haplotype frequencies were very similar in LABC patients and HC, the UTR-4 haplotype frequency was increased in prostate cancer patients compared to controls. Additionally, this haplotype was more frequent in women with an uneventful pregnancy and less frequent in women with recurrent miscarriage (32).

Regarding the clinical and prognostic significance of *HLA-G* 3’UTR haplotypes as indicators for a favorable or deleteriously disease status and outcome, conflicting results were reported: An age-adjusted logistic regression established UTR-4 as a risk factor in prostate cancer (34). In contrast to our study, the UTR-2 haplotype was reported to be a risk factor for colorectal cancer, whereas the UTR-4 haplotype resumed a protective role. In epithelial ovarian cancer, the homozygous UTR-1 genotype could be associated with metastases formation, whereas in our study, the presence of UTR-1 in LABC patients seems to be a beneficial factor for the response to NACT (24). In non-malignant situations, UTR-4 was reported to resume a protective role for the development of chronic kidney disease. In living-donor kidney transplantation, UTR-4 haplotype in donors and recipients was associated with occurrence of BK polyomavirus replication or nephropathy and with protection of antibody mediated rejection, whereas the donor UTR-2 haplotype was related to acute cellular or antibody-mediated rejection (28). Moreover, in our LABC patients’ cohort, the UTR-7 haplotype could be associated with high levels of circulating soluble as well as vesicular-bound HLA-G molecules, whereas in a recent study the UTR-5 and UTR-7 haplotypes were associated with low sHLA-G levels in French and Brazilian healthy individuals (31). Although high levels of HLA-GEV were found to be an independent parameter indicative for early relapse in LABC, the UTR-7 haplotype itself could not be established as a prognostic co-variate in LABC patients. This supports a recent observation that inhibition of effector immune cells *via* HLA-GEV is not restricted to cells expressing the ILT-2 receptors (35). Thus, all these partly contradictory associations of UTR haplotypes in physiological and pathological conditions underline a recent thesis that mechanisms regulating HLA-G expression can be distinctly active in different diseases (17). Indeed, the biologic function of the *HLA-G* 3’UTR haplotypes clearly depends on the presence or absence of regulatory elements targeting certain single nucleotides within the *HLA-G* 3’UTR. Thus, it is likely that, in cancer, the tumor location, phenotype, and heterogeneity may affect the functional consequence of certain haplotypes.

Concerning DTC, a recently published pooled analysis including 10.307 early BC patients has strengthened their prognostic significance as independent prognostic markers for OS, PFS, and distant disease-free survival. We recently demonstrated a phenotypic heterogeneity of DTC in early BC patients with regard to the expression of the chemokine receptor type 4 (CXCR4) as well as the transcription factor JUNB. Patients who harbored double-positive DTC (CXCR4- and JUNB-positive) showed an unfavorable clinical outcome (36). However, not all of the DTC have metastatic potential and not every patient with detectable DTC has a higher risk of relapse. Consequently, the additional analysis of factors that promote cancer invasiveness and metastatic progression, like the *HLA-G* 3’UTR haplotypes UTR-4, UTR-3, and UTR-7, might help to identify patients at a higher risk or with a favorable course more precisely. From the clinical point of view, it is particularly important that molecular genetic analysis of *HLA-G* 3’UTR haplotype analysis is less invasive than a bone marrow aspiration since it can be performed in blood and in the follow-up of the disease.

By the deduction of *HLA-G* 3’UTR haplotype to specific SNP, it became evident that the +3003C variant reflects the impaired clinical course of disease in UTR-4 carriers. This is in line with a study analyzing the potential use of *HLA-G* 3’UTR for prostate cancer prediction, in which the +3003C variant was suggested as a tag SNP for prostate cancer risk (33). Strikingly, two microRNAs, namely, miR-628-5p and miR-548q, have already been identified to target the segment encompassing the +3003 variant (13, 34). Binding of these microRNAs is most likely influenced by the presence of a certain nucleotide at this locus. In contrast, the protective role of UTR-2 could be deduced to the +3196G variant. In a study investigating kidney transplant recipients, stable allograft function was linked to the +3196CC genotype (37), suggesting an immunosuppressive role for this genotype. Of note, the +3196G variant is also characteristic for the rare UTR-8 as well as for UTR-10 haplotypes. However, both *HLA-G* 3’UTR haplotypes were not present at all in our cohort of LABC patients, whereas in the group of HC, the frequencies of these haplotypes were below 1% (0.2% and 0.4%, respectively).

The relevance of the gene polymorphisms of the *HLA-G* 3’UTR associated with LABC course/outcome is supported by the correlation between exosomal microRNA expression and disease stage of NACT-treated localized breast cancer (38). Here, distinct exosomal microRNAs allowed discrimination of localized from distant breast cancer patients and from healthy women (38). A combined study encompassing gene polymorphisms of the *HLA-G* 3’UTR and exosomal microRNAs could contribute to a better understanding of diagnosis, prognosis, and treatment response in breast cancer.

A limitation of the study might be the small number of patients included in this study to statistically discriminate between the different BC subtypes and the detected haplotypes. However, the results obtained for this group of LABC patients strengthen the fact that worse outcome seems to be multifactorial, underlining the requirement to perform a comprehensive analysis of the primary tumor as well as the liquid biopsy, which also includes circulating HLA-GEV to identify patients at higher risk to allow secondary treatment options. Regarding HLA-G and its cognate immune checkpoint receptor ILT-2 as targets for immunotherapeutic intervention, HLA-G-specific CAR-T cells are under development (39). Furthermore an anti-ILT-2 blocking antibody has been established and currently approved for a Phase I clinical trial (NCT04717375).

Combined, these data give evidence that the UTR-4 haplotype with its characteristic variant +3003C as well as the UTR-1 and UTR-2 haplotypes reflected by the +3187G or +3196G variant represent promising factors for the prognosis of disease outcome for LABC patients. Generally, OS and PFS in the cohort of patients presented here are improved, not only due to advanced therapeutic applications for these patients but also due to the fact that our patients were recommended an additional therapy with oral clodronate (2 × 520 mg per day for at least 2 years) in case of DTC positivity at primary diagnosis resulting in better outcomes (26). Thus, it has to be emphasized that the *HLA-G* 3’UTR variants or haplotypes may serve as a further promising parameter in the outcome of non-metastatic LABC patients.

## Data Availability Statement

The original contributions presented in the study are included in the article/supplementary material. Further inquiries can be directed to the corresponding author.

## Ethics Statement

The studies involving human participants were reviewed and approved by the Local Ethic Committee of the University Hospital in Essen, Germany. The patients/participants provided their written informed consent to participate in this study.

## Author Contributions

VR, ES, and SK-B conceived and designed research, performed the experiments, interpreted data, performed statistical analysis, wrote the initial draft, and read and approved the final article. LG performed experiments. RM and PH interpreted data, and read and approved the final article. A-KB, HR, RK, and OH collected and provided clinical data, interpreted data, and read and approved the final article. All authors contributed to the article and approved the submitted version.

## Funding

ES acknowledges support by the IFORES-Postdoctoral Excellence Program grant from the Medical Faculty of the University of Duisburg-Essen. We acknowledge support by the Open Access Publication Fund of the University of Duisburg-Essen. The funders had no role in the study design, data collection and analysis, decision to publish, or preparation of the manuscript.

## Conflict of Interest

Author SK-B is a consultant for Qiagen.

The remaining authors declare that the research was conducted in the absence of any commercial or financial relationships that could be construed as a potential conflict of interest.

## Publisher’s Note

All claims expressed in this article are solely those of the authors and do not necessarily represent those of their affiliated organizations, or those of the publisher, the editors and the reviewers. Any product that may be evaluated in this article, or claim that may be made by its manufacturer, is not guaranteed or endorsed by the publisher.
