# Supplementary material for: Systematic Evaluation of HLA-G 3’Untranslated Region Variants in Locally Advanced, Non-Metastatic Breast Cancer Patients: UTR-1, 2 or UTR-4 are Predictors for Therapy and Disease Outcome
Source: Front Immunol. 2022 Jan 12;12:817132. doi: 10.3389/fimmu.2021.817132 (PMC8790528; doi:10.3389/fimmu.2021.817132)
Supplement: Supplementary file 1 [file DataSheet_1.docx]

Additional file 1. Haplotype frequencies of polymorphic sites detected at the *HLA-G* 3’UTR in locally advanced breast cancer patients (n=142) and healthy donors (n=204) including 122 female and 82 male controls.

| **Haplotype** | **Frequency**  **female HC** | **Frequency**  **male HC** | **Frequency total HC** | **Frequency LABC** | **p_a_** | **OR (95% CI)** |
| --- | --- | --- | --- | --- | --- | --- |
| **UTR-1** | 34.0 | 35.4 | 34.6 | 29.6 | 0.180 | 0.79 (0.57 – 1.10) |
| **UTR-2** | 30.3 | 29.9 | 30.1 | 30.6 | 0.933 | 1.02 (0.74 – 1.43) |
| **UTR-3** | 7.4 | 9.8 | 8.3 | 7.0 | 0.567 | 0.83 (0.48 – 1.48) |
| **UTR-4** | 13.1 | 14.0 | 13.5 | 16.9 | 0.233 | 1.31 (0.85 – 1.98) |
| **UTR-5** | 2.9 | 2.4 | 2.7 | 2.5 | >0.99 | 0.91 (0.35 – 2.32) |
| **UTR-6** | 1.2 | 1.2 | 1.2 | 2.1 | 0.372 | 1.74 (0.51 – 5.02) |
| **UTR-7** | 4.9 | 3.7 | 4.4 | 6.0 | 0.308 | 1.38 (0.69 – 2.73) |
| **UTR-18** | 1.6 | 1.8 | 1.7 | 3.9 | 0.091 | 2.31 (0.91 – 5.98) |

Haplotype phasing was assessed by PHASE 2.1 software using default parameters. Only haplotypes with frequencies >1 % were listed. LABC – locally advanced breast cancer; HC – healthy controls; UTR – untranslated region. ^a^p-values were calculated by GraphPad Prism using two-sided Fisher’s exact test when evaluating haplotypes and Chi-Square test when assessing genotypes, alpha<0.05; OR, odds ratio

Additional File 2. Haplotype and genotype frequencies of the *HLA-G* 3’UTR in locally advanced breast cancer patients and healthy donors.

|  | **HC** | **LABC** |  | OR  (95% CI) |
| --- | --- | --- | --- | --- |
|  | N=204 (%) | N=142 (%) | *p^a^* |  |
| **UTR-1** |  |  |  |  |
| pos | 112 (54.9) | 71 (50.0) | 0.382 | 0.89 (0.69 – 1.14) |
| neg | 92 (45.1) | 71 (50.0) |  |  |
| Genotypes |  |  |  |  |
| UTR-1/UTR-1 | 29 (14.2) | 13 (9.2) | 0.334 |  |
| UTR-1/UTR-X | 83 (40.6) | 58 (40.8) |  |  |
| UTR-X/UTR-X | 92 (45.1) | 71 (50.0) |  |  |
| **UTR-2** |  |  |  |  |
| pos | 103 (50.4) | 73 (51.4) | 0.912 | 1.04 (0.67 – 1.60) |
| neg | 101 (49.5) | 69 (48.6) |  |  |
| Genotypes |  |  |  |  |
| UTR-2/UTR-2 | 20 (9.8) | 14 (9.9) | 0.985 |  |
| UTR-2/UTR-X | 83 (40.7) | 59 (41.5) |  |  |
| UTR-X/UTR-X | 101 (49.5) | 69 (48,6) |  |  |
| **UTR-3** |  |  |  |  |
| pos | 31 (15.2) | 19 (13.4) | 0.756 | 0.86 (0.47 – 1.60) |
| neg | 173 (84.8) | 123 (86.6) |  |  |
| Genotypes |  |  |  |  |
| UTR-3/UTR-3 | 3 (1.5) | 1 (0.7) | 0.768 |  |
| UTR-3/UTR-X | 28 (13.7) | 18 (12.7) |  |  |
| UTR-X/UTR-X | 173 (84.8) | 123 (86.6) |  |  |
| **UTR-4** |  |  |  |  |
| pos | 48 (23.5) | 41 (28.9) | 0.317 | 1.32 (0.79 – 2.16) |
| neg | 156 (76.5) | 101 (71.1) |  |  |
| Genotypes |  |  |  |  |
| UTR-4/UTR-4 | 7 (3.4) | 7 (4.9) | 0.507 |  |
| UTR-4/UTR-X | 41 (20.1) | 34 (23.9) |  |  |
| UTR-X/UTR-X | 156 (76.5) | 101 (71.1) |  |  |
| **UTR-5** |  |  |  |  |
| pos | 10 (4.9) | 7 (4.9) | 1.000 | 1.01 (0.38 – 2.81) |
| neg | 194 (95.1) | 135 (95.1) |  |  |
| Genotypes |  |  |  |  |
| UTR-5/UTR-5 | 1 (0.5) | 0 (0.00) | 0.746 |  |
| UTR-5/UTR-X | 9 (4.4) | 7 (4.9) |  |  |
| UTR-X/UTR-X | 194 (97.5) | 135 (95.1) |  |  |
| **UTR-6** |  |  |  |  |
| pos | 5 (2.5) | 6 (4.2) | 0.369 | 1.75 (0.51 – 5.14) |
| neg | 199 (0.97) | 136 (95.8) |  |  |
| Genotypes |  |  |  |  |
| UTR-6/UTR-6 | 0 (0.00) | 0 (0.00) | n.a. |  |
| UTR-6/UTR-X | 5 (2.5) | 6 (4.2) |  |  |
| UTR-X/UTR-X | 199 (97.5) | 136 (95.8) |  |  |
| **UTR-7** |  |  |  |  |
| pos | 16 (7.8) | 16 (11.3) | 0.36 | 1.49 (0.73 – 3.05) |
| neg | 188 (92.2) | 126 (88.7) |  |  |
| Genotypes |  |  |  |  |
| UTR-7/UTR-7 | 2 (0.9) | 1 (0.7) | 0.461 |  |
| UTR-7/UTR-X | 14 (6.9) | 15 (10.6) |  |  |
| UTR-X/UTR-X | 188 (92.2) | 126 (88.7) |  |  |
| **UTR-18** |  |  |  |  |
| pos | 7 (3.4) | 11 (7.7) | 0.088 | 2.36 (0.90 – 6.08) |
| neg | 197 (96.6) | 131 (92.3) |  |  |
| Genotypes |  |  |  |  |
| UTR-18/UTR-18 | 0 (0.00) | 0 (0.00) | n.a. |  |
| UTR-18/X | 7 (3.4) | 11 (7.7) |  |  |
| UTR-X/UTR-X | 199 (97.5) | 131 (92.3) |  |  |

CI – confidence interval; HC – healthy controls; LABC – locally advanced breast cancer; n.a. – not applicable; neg – negative; OR – odds ratio; pos – positive; UTR – untranslated region; UTR-X – every other UTR

^a^p-values were calculated by GraphPad Prism using two-sided Fisher’s exact test when evaluating haplotypes and Chi-Square test when assessing genotypes, alpha<0.05; OD, odds ratio
